# Effects of Antiplatelet and Anticoagulant Therapy on Emergency Surgical Outcomes in Orthopedic Trauma Patients With Cardiac Comorbidities

**DOI:** 10.7759/cureus.101322

**Published:** 2026-01-12

**Authors:** Yashar Mashayekhi, Mohamed Onsa, Gautam Chadalavada, Sara Baba-Aissa, Sahil Kakar, Sagar Vinayak, Shoaib Younas, Muhammad Zeeshan Akram, Muhammad Aqeel Qureshi, Muaz Shafique Ur Rehman

**Affiliations:** 1 Orthopedics, University Hospitals of Leicester, Leicester, GBR; 2 Trauma and Orthopedics, University Hospitals of Leicester, Leicester, GBR; 3 General Internal Medicine, Leicester Royal Infirmary, Leicester, GBR; 4 Orthopedics, Queen Elizabeth University Hospital, Glasgow, GBR; 5 Internal Medicine, School of Medicine, American University of Barbados, Bridgetown, BRB; 6 General Medicine, Basic Health Unit (BHU) Bindian, Khuiratta, PAK; 7 General Medicine, University Hospitals Birmingham NHS Foundation Trust, Birmingham, GBR; 8 Orthopedics, Mayo Hospital, Lahore, PAK; 9 Internal Medicine, Jinnah Hospital, Lahore, PAK

**Keywords:** anticoagulant therapy, antiplatelet, non-surgical orthopedics, patients, therapy

## Abstract

Background: Emergency orthopedic trauma surgery in patients with cardiac comorbidities presents a major clinical challenge due to the widespread use of antiplatelet and anticoagulant therapy.

Objective: To evaluate the impact of antiplatelet and anticoagulant therapy on perioperative bleeding, surgical timing, postoperative complications, and mortality in orthopedic trauma patients with cardiac comorbidities.

Methods: This retrospective cross-sectional study was conducted at Jinnah Hospital, Lahore, from June 2024 to June 2025. It included 246 patients with cardiac comorbidities who underwent emergency orthopedic trauma surgery. Emergency surgery was defined as operative fixation required within 72 hours of injury based on pain severity, fracture instability, neurovascular risk, or potential displacement. Data were collected on demographics, comorbidities, type of antithrombotic therapy, fracture patterns, surgical timing, intraoperative blood loss, transfusion requirements, postoperative complications, length of stay, and in-hospital mortality.

Results: The mean age of patients was 64.2±11.8 years, with 60.6% being male. Hip fractures were the most common injury (65.8%). Antiplatelet therapy was used in 55.7% of patients, anticoagulants in 31.7%, and combined therapy in 12.6%. Patients on dual antiplatelet therapy and anticoagulants had significantly greater blood loss (742±235 ml and 689±228 ml, respectively) and higher transfusion requirements compared with aspirin-only patients. Surgical delay was longest in patients on direct oral anticoagulants (56.2±14.9 hours). Postoperative complications included wound hematoma (13.8%), infection (7.7%), thromboembolic events (6.9%), and reoperation (4.5%). Overall mortality was 8.5%, highest among patients on dual antiplatelet therapy (11.7%). Logistic regression identified dual antiplatelet therapy (OR 2.4, 95% CI 1.2-4.8), anticoagulant therapy (OR 1.9, 95% CI 1.1-3.6), surgical delay >48 hours (OR 2.8, 95% CI 1.5-5.1), and age >70 years (OR 2.2, 95% CI 1.1-4.3) as independent predictors of adverse outcomes.

Conclusion: Antiplatelet and anticoagulant therapy significantly influence emergency surgical outcomes in orthopedic trauma patients with cardiac comorbidities. These findings support the need for standardized multidisciplinary perioperative protocols including early cardiology input, structured antithrombotic management pathways, and expedited surgical planning to optimize outcomes in this high-risk population.

## Introduction

Orthopedic trauma surgery in patients with coexisting cardiac disease is no longer a rare clinical experience but a pressing daily issue in the majority of emergency departments across the globe [1]. As more patients present with cardiovascular diseases, especially coronary artery disease and atrial fibrillation, and are already taking long-term antiplatelet or anticoagulant therapy, a substantial percentage of orthopedic trauma patients present using these medications [2]. These treatments, though necessary to decrease cardiovascular morbidity and mortality, add a nuance of risk and uncertainty in instances where a prompt surgical procedure is required [3]. The surgeon’s dilemma is often urgent: either to proceed with early fixation and risk massive intraoperative bleeding, or to postpone surgery until the drugs are cleared, thereby subjecting the patient to an increased risk of thromboembolic events, cardiac decompensation, or worsened orthopedic outcomes. This dilemma is particularly pronounced in situations such as hip and femoral fractures, where it has been consistently shown that delays of more than 24-48 hours significantly increase mortality, length of hospitalization, and complications such as pneumonia, venous thromboembolism, and functional decline [4]. Simultaneously, premature cessation of antiplatelet therapy following coronary stenting, particularly within the first 30 days, can trigger acute stent thrombosis, an event associated with a mortality rate exceeding 40% [5]. Likewise, withholding anticoagulation in patients with atrial fibrillation, mechanical prosthetic valves, or recent venous thromboembolism places them at increased risk of systemic embolism, stroke, or valve thrombosis. This represents a high-risk trade-off between hemorrhagic and thrombotic complications and is a central challenge in the perioperative management of these high-risk patients [6]. Pharmacologically, antiplatelet and anticoagulant drugs differ in their mechanisms of action, although they share a common final effect: inhibition of hemostasis. Aspirin irreversibly inhibits platelet cyclooxygenase, while clopidogrel and newer P2Y12 inhibitors prevent platelet activation by adenosine diphosphate (ADP). Anticoagulants modulate the coagulation cascade, with warfarin inhibiting vitamin K-dependent clotting factors, heparin activating antithrombin III, and direct oral anticoagulants (DOACs) directly inhibiting thrombin or factor Xa [7].

The perioperative issues associated with each of these classes are different: aspirin can exacerbate blood loss but is widely available; clopidogrel can raise the risk of transfusion and hematoma; warfarin must be reversed with vitamin K or prothrombin complex concentrate; DOACs are convenient to use in routine practice, but they make it difficult to time surgery because no one can predict their clearance; and specific anticoagulants cannot be reversed [8]. The amount of blood loss alone does not determine the clinical impact of surgery conducted under active antithrombotic treatment. The formation of hematomas may blur the operating field, extend the duration of surgery, and complicate anesthesia. Hematomas and wound ooze are risk factors for postoperative infection, wound dehiscence, reoperation, and delayed rehabilitation [9]. In contrast, many strategies to reduce bleeding, including discontinuation or reversal of therapy, tend to expose the patient to ischemic cardiac events, perioperative myocardial infarction, or thromboembolic stroke [10]. It is debated whether one should use bridging protocols with low-molecular-weight heparin, as it has been reported to increase bleeding and fail to significantly reduce thrombotic events, especially in emergency trauma settings. System-level and logistical problems add to the complexity of this clinical problem [11]. Laboratory tests to measure DOAC activity are not available in most hospitals, including resource-constrained hospitals; reversal agents might be inaccessible; and team consultation between orthopedic surgeons, cardiologists, and anesthesiologists might be impossible under time pressure [12]. In the last 10 years, various observational studies and meta-analyses have tried to explain how best these patients are handled; however, the evidence is still highly fragmented and usually generalized from cohorts of elective surgery as opposed to emergency trauma. The available data indicate that surgery cannot be postponed in cases of aspirin monotherapy, whereas dual antiplatelet therapy is more dangerous [13].

Objective

To evaluate the impact of antiplatelet and anticoagulant therapy on perioperative bleeding, surgical timing, postoperative complications, and mortality in orthopedic trauma patients with cardiac comorbidities.

## Materials and methods

This retrospective cross-sectional study was conducted at Jinnah Hospital, Lahore, from June 2024 to June 2025. It included 246 patients with cardiac comorbidities who underwent emergency orthopedic trauma surgery. This study included patients aged 18 years and above who presented with acute orthopedic trauma necessitating emergency surgical intervention. Patients with documented cardiac comorbidities such as ischemic heart disease, atrial fibrillation, valvular disease, or heart failure were eligible for inclusion. Patients who were currently on established antiplatelet or anticoagulant therapy at the time of trauma were also included in the study. Patients younger than 18 years, those with polytrauma requiring neurosurgical or abdominal surgical prioritization, and patients with pre-existing coagulopathy unrelated to antiplatelet or anticoagulant use were excluded. Furthermore, patients with incomplete medical or surgical records were excluded from the study to ensure accurate and comprehensive data collection. Non-probability consecutive sampling was employed to recruit patients meeting the inclusion criteria.

Data collection

Data were extracted from a variety of hospital records, including surgical notes, anesthesia charts, and laboratory files. The collected variables included demographic details such as age and gender, type of orthopedic injury, and type of cardiac comorbidity. Information regarding antiplatelet or anticoagulant therapy, including the specific agent, dose, and duration, was also recorded. Additionally, data on reversal or bridging strategies used, time-to-surgery, estimated intraoperative blood loss, transfusion requirements, and operative duration were captured. Postoperative outcomes, including complications such as hematoma, wound infections, thromboembolic events, and myocardial injury, were documented. The length of hospital stay and in-hospital mortality data were also included in the analysis. Intraoperative blood loss was quantified using suction measurements (subtracting irrigation volume), sponge counts, and postoperative drain output. Postoperative complications were verified through chart documentation using predefined diagnostic criteria, including imaging-confirmed thromboembolic events (Doppler ultrasound or CT pulmonary angiography), microbiologically or clinically diagnosed wound infections, hematoma documented by surgical assessment, and myocardial injury confirmed by ECG and troponin testing. In-hospital mortality was defined as any death from admission until discharge, confirmed through medical records, nursing notes, and mortality logs.

Data analysis

All data were entered into SPSS version 26 (IBM Corp., Armonk, NY). Continuous variables (age, blood loss, and time-to-surgery) were presented as mean ± standard deviation. Categorical variables (type of fracture, drug category, and presence of complications) were expressed as frequencies and percentages. Group comparisons (antiplatelet vs. anticoagulant, single vs. dual therapy, and reversal vs. non-reversal) were analyzed using independent t-tests or ANOVA for continuous variables and the chi-square test for categorical variables. A p-value <0.05 was considered statistically significant.

## Results

The study consisted of 246 patients with a mean age of 64.2±11.8 years, showing that the majority of patients were older adults. Males accounted for 60.6% of the sample. Ischemic heart disease was the most common cardiac comorbidity (53.6%), followed by atrial fibrillation (28.9%), heart failure (11.8%), and prosthetic heart valves (5.7%). Hypertension (66.7%) and diabetes mellitus (45.5%) were frequently associated conditions. Regarding therapy, aspirin monotherapy was the most common regimen (35.0%), while 20.7% were on dual antiplatelet therapy. Warfarin and DOAC use were equally prevalent (15.9% each), and 12.6% were receiving a combination of antiplatelet and anticoagulant therapy (Table 1).

**Table 1:** Baseline characteristics of patients (n=246) DOAC, direct oral anticoagulant

Characteristics	Value
Age (years), mean ± SD	64.2±11.8
Male gender, n (%)	149 (60.6%)
Ischemic heart disease, n (%)	132 (53.6%)
Atrial fibrillation, n (%)	71 (28.9%)
Heart failure, n (%)	29 (11.8%)
Prosthetic heart valve, n (%)	14 (5.7%)
Hypertension, n (%)	164 (66.7%)
Diabetes mellitus, n (%)	112 (45.5%)
Therapy type	
Aspirin only	86 (35.0%)
Dual antiplatelet therapy (aspirin + clopidogrel)	51 (20.7%)
Warfarin	39 (15.9%)
DOACs (apixaban, rivaroxaban, dabigatran)	39 (15.9%)
Combination (antiplatelet + anticoagulant)	31 (12.6%)

Hip fractures represented the majority of cases (65.8%), followed by femoral shaft (17.1%) and tibial fractures (11.0%). The mean time from admission to surgery was 42.6±16.4 hours. Patients on antiplatelet therapy underwent surgery sooner (36.5±12.8 hours), while those on warfarin (48.1±15.2 hours) and DOACs (56.2±14.9 hours) experienced significant delays, reflecting caution in perioperative management (Table 2).

**Table 2:** Fracture patterns and surgical timing DOAC, direct oral anticoagulant

Variable	Value
Hip fracture	162 (65.8%)
Femoral shaft fracture	42 (17.1%)
Tibial fracture	27 (11.0%)
Other fractures	15 (6.1%)
Mean time to surgery (hours)	42.6±16.4
Time to surgery: antiplatelet	36.5±12.8
Time to surgery: warfarin	48.1±15.2
Time to surgery: DOACs	56.2±14.9

The mean intraoperative blood loss was 624±210 mL, with the highest losses seen in patients on dual antiplatelet therapy (742±235 mL) and DOACs (689±228 mL). Transfusion requirements were greater in patients on anticoagulants (62.8%) compared to those on antiplatelets (44.5%). Postoperative complications included wound hematoma (13.8%), surgical site infection (7.7%), reoperation (4.5%), and thromboembolic events (6.9%) (Table 3).

**Table 3:** Intraoperative and postoperative outcomes DOAC, direct oral anticoagulant

Outcome	Value
Mean intraoperative blood loss (mL)	624±210
Blood loss in dual antiplatelet therapy (mL)	742±235
Blood loss in DOAC patients (mL)	689±228
Transfusion required: anticoagulants	49 (62.8%)
Transfusion required: antiplatelets	61 (44.5%)
Wound hematoma	34 (13.8%)
Surgical site infection	19 (7.7%)
Reoperation	11 (4.5%)
Thromboembolic events	17 (6.9%)

The mean length of hospital stay was 9.8±4.6 days, with patients on anticoagulants staying longer (11.6±5.2 days) than those on antiplatelets (8.7±4.1 days). Overall mortality was 8.5% (n=21). The highest mortality was observed among patients on dual antiplatelet therapy (11.7%) and anticoagulants (10.3%), compared with 5.8% in aspirin-only patients (Table 4).

**Table 4:** Hospital stay and mortality

Variable	Value
Mean hospital stay (days)	9.8±4.6
Hospital stay: anticoagulant group	11.6±5.2
Hospital stay: antiplatelet group	8.7±4.1
Overall mortality	21 (8.5%)
Mortality: dual antiplatelet therapy	6 (11.7%)
Mortality: anticoagulant therapy	8 (10.3%)
Mortality: aspirin-only	5 (5.8%)

Multivariate logistic regression identified dual antiplatelet therapy (OR 2.4, p=0.014), anticoagulant therapy (OR 1.9, p=0.038), surgical delay beyond 48 hours (OR 2.8, p=0.006), and age greater than 70 years (OR 2.2, p=0.027) as independent predictors of postoperative complications and mortality (Table 5).

**Table 5:** Predictors of adverse outcomes (multivariate logistic regression)

Predictor	OR	95% CI	P-value
Dual antiplatelet therapy	2.4	1.2-4.8	0.014
Anticoagulant therapy	1.9	1.1-3.6	0.038
Surgical delay >48 hours	2.8	1.5-5.1	0.006
Age >70 years	2.2	1.1-4.3	0.027

## Discussion

This paper draws attention to the multifactorial relationship between anticoagulant or antiplatelet treatment and surgical outcomes in the emergency setting for orthopedic patients with cardiac comorbidities. Our sample (n=246) revealed that although antithrombotic therapy is vital for cardiovascular protection, it is linked to increased perioperative bleeding, longer preoperative times, more transfusions, and increased death rates, especially in patients receiving dual antiplatelet therapy or oral anticoagulants. We found that, in agreement with previous studies, patients receiving dual antiplatelet therapy are more prone to intraoperative loss and postoperative complications than patients receiving single-agent therapy. Our cohort showed a mean blood loss of 742 mL in the dual antiplatelet group, which was significantly higher than in patients receiving aspirin only. This is congruent with existing literature indicating up to two times higher transfusion rates in patients who underwent dual platelet inhibition. Likewise, patients receiving DOACs and warfarin experienced delays in surgical intervention, which is evidence of clinical precautions in perioperative management for this population [14]. Prior reports have also noted long time-to-surgery among anticoagulated patients, and delays are directly proportional to morbidity and mortality. This research supports the fine line between bleeding and thrombosis in this at-risk group. Whereas continuation of treatment increases the chances of hematoma, wound infection, and transfusion, termination or reversal puts patients in danger of thromboembolic complications. Thromboembolic events were common in our cohort, with myocardial infarction and stroke being the most frequent [15]. This occurrence is similar to previous observational studies reporting perioperative cardiac events between 5% and 10% in similar populations. The fact that mortality rates are high in patients placed on anticoagulation (10.3%) and dual antiplatelet therapy (11.7%) underlines the antagonistic risks of dealing with these interventions in the trauma environment. Surgical delay became an important predictive factor of poor outcomes [16]. Patients who had surgery beyond 48 hours had high complication and mortality rates. Surgical delay longer than 48 hours is an independent predictor of adverse events, as proven by logistic regression (OR 2.8, 95% CI 1.5-5.1). This observation repeats an established principle in orthopedic trauma literature that early fixation, especially in hip fractures, is closely linked with diminished morbidity and mortality. Nevertheless, the presence of antithrombotic treatment often encourages clinicians to postpone surgery until coagulation parameters are restored or remedial measures are implemented. We have evidence of the poor clinical outcomes of these delays, and it is clear that hospital-wide measures are necessary to improve safe surgical care delivery in anticoagulated patients [17]. This information can be of great significance to multidisciplinary care. The evidence indicates that aspirin monotherapy does not necessarily justify surgical delay because the mortality and risk of bleeding were lowest in this group. Conversely, patients on dual antiplatelet therapy or anticoagulant therapy need specific approaches, which may include the application of reversal therapy, platelet transfusion, or intraoperative supplements such as tranexamic acid. Repeated cardiology and anesthesiology interventions are also important to balance the risks of bleeding against the risks of cardiac or thromboembolic events. Additionally, the substantial increase in hospitalization for anticoagulated patients demonstrates a significant resource waste from postponed surgery and a higher occurrence of complications [18].

Strengths and limitations

The study’s main strength lies in its sizable real-world cohort of high-risk cardiac patients requiring urgent orthopedic surgery, an understudied population where evidence is most needed. The detailed categorization of antithrombotic therapy provides practical insights for clinical decision-making. However, several limitations must be acknowledged. Confounding may persist due to variability in surgeon expertise, injury severity, and perioperative protocols across teams. Reversal strategies were physician-dependent and not standardized, limiting causal interpretation. Anticoagulation data were incomplete in some cases, particularly with DOAC timing. Resource constraints, including limited availability of reversal agents and delayed laboratory turnaround, may have influenced surgical timing and outcomes. Complications and mortality were limited to in-hospital events, preventing assessment of post-discharge sequelae. The single-center retrospective design also limits external generalizability.

## Conclusions

It is concluded that antiplatelet and anticoagulant therapy were associated with higher perioperative risk in emergency orthopedic trauma surgery, and these associations remained significant after adjustment for key clinical factors. The findings suggest that aspirin monotherapy generally does not warrant surgical delay, whereas patients on dual antiplatelet or anticoagulant therapy benefit from structured perioperative planning, including early cardiology and anesthesia input and rapid reversal pathways when appropriate. Surgical delay beyond 48 hours independently increased complications and mortality, indicating that timing guidelines should prioritize minimizing avoidable delays. As this study captures only in-hospital outcomes, longer-term risks remain unknown. Further prospective studies are needed; however, the results support implementing multidisciplinary protocols to improve safety and reduce delays in this high-risk population.
